# Post-stroke Rehabilitation of Severe Upper Limb Paresis in Germany – Toward Long-Term Treatment With Brain-Computer Interfaces

**DOI:** 10.3389/fneur.2021.772199

**Published:** 2021-11-18

**Authors:** Cornelius Angerhöfer, Annalisa Colucci, Mareike Vermehren, Volker Hömberg, Surjo R. Soekadar

**Affiliations:** ^1^Clinical Neurotechnology Lab, Department of Psychiatry and Neurosciences, Charité–Universitätsmedizin Berlin, Berlin, Germany; ^2^Department of Neurology, SRH Gesundheitszentrum Bad Wimpfen GmbH, Bad Wimpfen, Germany

**Keywords:** brain-computer interface, severe upper limb paresis, neurorehabilitation, long-term treatment, neurotechnology

## Abstract

Severe upper limb paresis can represent an immense burden for stroke survivors. Given the rising prevalence of stroke, restoration of severe upper limb motor impairment remains a major challenge for rehabilitation medicine because effective treatment strategies are lacking. Commonly applied interventions in Germany, such as mirror therapy and impairment-oriented training, are limited in efficacy, demanding for new strategies to be found. By translating brain signals into control commands of external devices, brain-computer interfaces (BCIs) and brain-machine interfaces (BMIs) represent promising, neurotechnology-based alternatives for stroke patients with highly restricted arm and hand function. In this mini-review, we outline perspectives on how BCI-based therapy can be integrated into the different stages of neurorehabilitation in Germany to meet a long-term treatment approach: We found that it is most appropriate to start therapy with BCI-based neurofeedback immediately after early rehabilitation. BCI-driven functional electrical stimulation (FES) and BMI robotic therapy are well suited for subsequent post hospital curative treatment in the subacute stage. BCI-based hand exoskeleton training can be continued within outpatient occupational therapy to further improve hand function and address motivational issues in chronic stroke patients. Once the rehabilitation potential is exhausted, BCI technology can be used to drive assistive devices to compensate for impaired function. However, there are several challenges yet to overcome before such long-term treatment strategies can be implemented within broad clinical application: 1. developing reliable BCI systems with better usability; 2. conducting more research to improve BCI training paradigms and 3. establishing reliable methods to identify suitable patients.

## Introduction

Stroke is a leading cause for long-term disability and often results in poor quality of life (1). In 2017, there were around 260,000 in-patient cases of stroke registered in German hospitals (2). While the incidence in Germany mostly remained constant (3), the probability to survive an acute stroke significantly improved over the last two decades (4). With a growing number of stroke survivors, the number of patients facing post-stroke impairments is increasing. Besides impairments in cognition, speech, mood regulation or sexual function, loss of motor function, especially in the upper extremity, is a severe burden after stroke.

Upper limb impairment occurs in approximately 80% of stroke survivors (5). Here, the initial severity of motor impairment often predicts chances for recovery (6). While there is a good chance for full recovery from mild paresis, this is less likely for severe upper limb paresis (7). In 30–50% of all stroke survivors, the affected arm is still severely impaired 6 months after stroke (8, 9). This group face immense difficulties in performing activities of daily living (ADLs) and must often rely on family support or caregivers (10). Given the rising burden of stroke, there is a pressing need for innovative tools that foster successful restoration of motor function.

## Rehabilitation of Severe Upper Limb Paresis

Constraint-induced movement training (CIMT) represents an effective motor intervention in upper limb rehabilitation (11–13). However, as voluntary wrist and finger extension is a minimum requirement for CIMT, it is only applicable in a limited number of stroke patients, usually excluding those with severe upper limb paresis (14). The treatment repertoire for patients with severe motor impairment is small. Classical physiotherapy is often applied as standard therapy. Mirror therapy has shown to improve arm and hand function (13, 15, 16). In mirror therapy, the unimpaired hand is observed in a mirror projected onto the side of the impaired hand. Since such training does not require remaining motor function in the paretic limb, it is recommended as a complementary approach for stroke survivors with severe arm paralysis (17). Arm-BASIS-Training (ABT) is a method of impairment-oriented training that aims at restoring motoric innervation by selectively training specific arm movements (e.g., shoulder joint movements, elbow joint movements, wrist joint movements), exclusively designed for the rehabilitation of severe upper limb paresis (18). In a systematic review by Urton et al. (19), ABT was the only intervention that improved arm mobility in addition to standard physiotherapy, receiving recommendation grade A. The use of external devices (robotics) allows to train movements with high repetition rates which cannot be performed by the patient independently. It was shown that robotic-aided therapy can improve arm function, force and mobility in stroke patients with severe upper limb paresis (20, 21).

While there are well-evidenced motor interventions for mild paralysis, successful rehabilitation of severe upper limb paresis is still an unsolved problem: For classical physiotherapy, evidence of efficacy is not convincing (12, 22, 23). Impairment-oriented training was shown to improve selective arm mobility, but not arm function (18). Robotics-guided therapy is useful in the subacute stage, but there is still a controversy whether also chronic patients benefit from this therapy. Furthermore, it is not clear yet whether it can also improve ADL skills and thus reduce or prevent the necessity of long-term care (24, 25). The same has also not been conclusively clarified for mirror therapy (16, 26). With the lack of standardized and convincing treatment options, there is high demand to find new strategies to restore severe upper limp paresis in stroke survivors.

## Brain-Computer Interface and Brain-Machine Interface

Brain computer interfaces (BCIs) use the modulation of neurometabolic or neuroelectric signals to control external devices (27). By analyzing changes in brain activity, BCI technology can convert the user's intention into control commands of digital devices or tools usually delivering some form of sensory feedback. A sub-form of BCI systems enabling volitional control of machines, e.g., exoskeletons or prostheses, are usually termed brain-machine interfaces (BMIs). The most established BCI paradigms are based on electroencephalography (EEG), due to its low cost and easy handling (28). In BCI and BMI applications for motor rehabilitation, EEG is typically used to record the sensorimotor rhythm (SMR), generated by neuronal cell populations of the sensorimotor cortex (29). During motor attempt or imagination, power of SMR decreases and this modulation, called event-related-desynchronization (ERD), can be translated into a control command for an external device. Since even stroke survivors with severe chronic motor deficits can modulate their SMR (30), and no actual physical movement is required to control BCI-based devices, they represent a promising rehabilitation strategy for stroke patients with severe upper limb paresis.

BCI technology addresses important physiological fundamentals of neurorehabilitation. Based on Hebbian learning principles, such systems can induce neuroplasticity by effectively coupling efferent brains signals to afferent input (e.g., caused by a closing exoskeleton) (31). This way, neural assemblies are activated in an associative manner, strengthening cortical connections, as evidenced by increased motor evoked potentials (MEP) in stroke survivors using a BCI (32). Linking central motor output to peripheral input in real-time closes the sensorimotor feedback loop, which can promote the integration of affected corticospinal connections (33) and foster voluntary motor control (34). Moreover, BCIs improve the ability to activate affected brain areas by visualizing changes in brain activity in real-time (a paradigm termed neurofeedback) (34). This was shown to result in greater involvement of the ipsilesional hemisphere compared to random feedback (35). Apart from influencing neurophysiological parameters reflecting neuroplasticity, BMI applications also showed to induce functional improvement (36–39).

Although BCI-based approaches appear to be promising for upper limb rehabilitation, sufficient evidence for broad clinical application is still lacking and more research is needed. However, conducting studies according to principles of evidence-based medicine (EBM) is often difficult due to the heterogeneity of stroke patients. Further, BCI-based motor therapy involves far more variables than just dose and timing, and double blinding is difficult to implement. Therefore, Coscia et al. (40) suggest investigating BCI applications in personalized longitudinal studies in contrast to randomized controlled trials (RCTs). In such a design, patients receive specific treatment until a functional plateau is reached, before continuing with another intervention. The primary aim of such approach is to improve the individual outcome in patient populations that usually have poor prospects for recovery and rarely show spontaneous remission (40). In this context, it is crucial to find long-term treatment strategies that can be well integrated throughout the different phases of neurorehabilitation. Beyond research, such strategies are of great importance for clinicians who apply BCI-based therapy as early-adopters to expand treatment options. Since BCI-based therapy addresses several aspects of motor learning (neurofeedback, training of repetitive tasks, active behavior in therapy), this provides good reason for early application (41) –especially as there are only few alternatives for restoring severe upper limb paresis in stroke patients.

## Bci-Based Long-Term Treatment Strategies in Germany

Thanks to its various applications, BCI-based therapy can be embedded in many stages of stroke rehabilitation. Here, based on a literature review, we derived the best strategies to facilitate and accelerate the integration of BCI-based therapy into the German process of neurorehabilitation. We include studies that assess the impact of BCI-based interventions on motor recovery of stroke patients with severe upper limb paresis, either in the subacute (<6 months) or chronic stage (>6 months). Study outcomes were either functional scores and/or neurophysiological parameters. Case studies and studies with small sample size (<10 participants) were not included. In Germany, the process of neurological rehabilitation is divided into 6 phases (A to F): Phase A represents the emergency care on a stroke unit, followed by phase B which is equivalent to early rehabilitation. In Phase C, patients can actively participate in rehabilitation interventions, however, there is still high demand for medical treatment. In Phase D, patients receive medical rehabilitation in specialized rehabilitation centers (Phase D), which corresponds to the concept of post hospital curative treatment. In Phase E, outpatient occupational reintegration is pursued and, in case long-time care is necessary, Phase F is initiated to support and maintain function (42).

In acute care and early rehabilitation (Phase A and B), BCI-based applications often cannot be applied due to patient's reduced vigilance and impaired cooperativity (43). However, as soon as active participation is possible (Phase C), BCI-based training can be started. At the beginning, it is advisable to familiarize patients with BCI technology by providing neurofeedback on SMR modulation related to motor imagery (MI). As shown by Pichiorri et al. (35) and Mihara et al. (44), BCI assisted neurofeedback on MI can contribute to functional recovery and enhance neural connectivity on the affected brain hemisphere in subacute stroke patients. Compared to visual feedback, somatosensory and/or proprioceptive feedback seems advantageous in rehabilitation (45). Furthermore, daily MI-based neurofeedback training has been shown to improve SMR control (46), facilitating the control of BMI motor interventions later on. Since patients do not have to be mobilized for neurofeedback training, this can be applied on bedside, increasing accessibility. In phase C, the patient's state of health is often still unstable, and exhaustion caused by intensive motor therapy could do more harm than good.

Although there are no RCTs comparing early vs. late onset, a general recommendation is that the earlier motor interventions start, the better (17). Thus, BCI therapy should advance once SMR control is well established, and the patient's clinical condition allows. In a next step, EEG-based BMI systems can be used to control robotic therapy devices to effectively link repetitive exercise training with cortical motor output. Ang et al. (36) showed that such a BMI system coupled with the MIT-Manus, a robotic arm that initiates and guides upper limb movements, enhances arm and hand function in stroke patients with severe upper limb paresis. It was reported that such therapy is well tolerated by patients and no adverse effects occur, making it an effective and safe method for upper limb rehabilitation. As BMI robotic training requires expensive equipment and specialized staff, it seems best to apply such therapy in specialized rehabilitation centers (Phase D). Here, subacute stroke patients come together in large numbers, guaranteeing maximum capacity utilization and effective use. Besides brain controlled robotic therapy, BCIs can be applied for exoskeleton control and FES. Exoskeletons are portable devices that support or completely imitate movement, while FES uses electrical pulses to stimulate muscle contractions, enabling the movement of paralyzed limbs. Biasiucci et al. (37) showed that BCI-driven FES can reduce severe upper limb impairment in subacute stroke patients. Most notably, functional improvement prevailed 6 months after therapy, making it a promising strategy for a curative treatment approach (Phase D).

There are good reasons to continue BCI therapy in chronic stroke patients that already showed some degree of motor recovery. In patients with initial hemiparesis but returning arm control, EEG-based hand exoskeletons can be used to restore hand function. It was shown that the application of a BCI-driven hand exoskeleton in chronic stroke patients reduces complete finger paralysis (39), improves grip function (47) and results in an increased use of the paralyzed hand in ADL tasks (48). A major advantage of BCI-based exoskeleton control is that it allows patients to perform grasping movements with their paralyzed hand and enables them to perform bimanual tasks in training sessions (e.g., eating with cutlery), counteracting the learned non-use of the paralyzed limb. The high relation of such exercises to everyday life situations promotes patient's motivation to continue therapy. This is especially important as depression and reduced drive are severe complications following stroke (49). Considering the reduced effort needed in comparison to robotic-guided therapy, the applicability in chronic patients and the practicability in bimanual tasks, BMI hand exoskeleton training is well suited for outpatient occupational therapy (Phase E). This way, a continuous treatment is pursued even after medical rehabilitation (Phase D) is completed.

In case no sufficient improvement can be achieved, nursing or family care (Phase F) is often unavoidable for patients with remaining severe upper limb impairment. Although the rehabilitative potential might be exhausted in these cases, the BCI approach can be used to compensate for lost function by controlling assistive devices, e.g., exoskeletons or robotic prostheses (assistive BCIs). Bundy et al. (50) have demonstrated the feasibility of a BCI-based hand exoskeleton for home use in chronic stroke patients, using EEG signals of the unaffected hemisphere. Applied in a home-based setting, assistive BCIs allow patients to perform important ADLs such as eating and drinking independently (51). This way, patients become less reliant on family or caregiver support and can regain quality of life.

## Current Challenges and Limitations

Attaching and calibrating BCI systems currently requires the support of specially trained personnel and takes considerable amount of time (52). Since time is a scarce commodity in clinical practice, preparation for BCI-based therapy must become quick and easy, e.g., through intuitive graphical user interfaces and fast automatic calibration systems. For assistive BCIs applied in a home-based setting, the need of a supervisor contradicts the idea of giving back independency. Although improvements have been made to enhance practicability by implementing veto commands (53), establishing hybrid control paradigms (54) and developing user-friendly EEG systems (55, 56), assistive BCI systems that can be applied by the user without any external help are still missing. Another drawback of BCI-based interventions lies in the high acquisition costs of the technical devices. BCI-based therapy must yet prove its clear effectiveness to justify the high expenses, otherwise it will not find entrance in clinical practice. Consequently, efforts need to be made to drive technical development, lower cost of production and conduct studies that thoroughly investigate the benefit of BCI technology (33).

BCIs can fail to detect modulation of brain activity in up to 20% of individuals (57). While some speak of BCI-illiteracy, Vidaurre et al. (58) showed that machine learning approaches can considerably reduce the number of BCI “illiterates.” This should encourage researchers to improve BCI classification and paradigms rather than to attribute the fault to the user (59). Current training approaches for brain modulation often seem inappropriate, e.g., lacking clear instructions, specific learning objectives and meaningful feedback. This affects BCI robustness and requires new training procedures (60). Stroke patients in particular may have difficulty modulating their cortical activity due to the brain lesion, potentially leading to frustration when trying to control BCI-based devices (61). In this context, it may be advantageous to individually adapt session time of BCI-based therapy since fatigue and lack of concentration can affect BCI control substantially (62, 63). While heart rate variability (HRV) (64) and task-related theta-band activity (65) have been proposed as possible biomarkers to monitor patient's mental capacity during BCI sessions, there is still much work needed to establish parameters that reliably anticipate mental workload and fatigue. Overall, more research assessing user experience with BCIs needs to be conducted, especially considering hedonic quality aspects such as motivation and frustration since they play an important role in stroke rehabilitation (66). Also, neuroplasticity mechanisms induced by BCI-based application need to be better understood, with regards to so far less studied aspects such as wide-spread neural networks and cortical excitability (67, 68).

For motor interventions to be most effective, it has been suggested to move from a “one-fits-all” approach toward customized rehabilitative interventions (69). In this context, identification of stroke patients that will benefit most from BCI therapy needs to be realized. So far, it has been shown that functional connectivity assessed by functional magnetic resonance imaging (fMRI) predicts motor recovery and outcome of sensorimotor function scores (70, 71). In this light, it may be reasonable to use fMRI to determine patient's potential for motor recovery and his/her eligibility to benefit from BCI therapy (33). Sannelli et al. (72) suggested to group BCI users according to their calibration and feedback performance to select subjects and allow customized training. Overall, however, it is not yet possible to precisely estimate how many stroke survivors can directly benefit from BCI-based therapy. Only largescale, longitudinal clinical studies will provide the necessary data to infer the underlying mechanisms of BCI-related recovery and to identify predictors of individual BCI-training response.

## Conclusions

With its capability to advance motor recovery, BCI-based motor interventions provide a good argument to be implemented in stroke rehabilitation in Germany. BCI-based neurofeedback training is well suited to promote neural and functional recovery in early rehabilitation. BCI-based motor interventions showed efficiency in both subacute and chronic stroke patients, making them suitable for post hospital curative treatment and outpatient occupational therapy. Once a plateau in motor recovery is reached, assistive BCI-based devices can be applied in a home setting to improve quality of life. Put together, BCIs represent a promising approach for long-term treatment of severe upper limb paresis in stroke patients. Nevertheless, there are still some obstacles to overcome before BCI-based therapies can be applied in routine clinical practice.

## Author Contributions

CA and SRS conceived the topic. CA conceptualized the manuscript. CA took the lead in writing the manuscript and was supported by AC, MV, VH, and SRS. All authors provided critical feedback and helped shape the research idea and manuscript, reviewed the results, and approved the final version of the manuscript.

## Conflict of Interest

The authors declare that the research was conducted in the absence of any commercial or financial relationships that could be construed as a potential conflict of interest.

## Publisher's Note

All claims expressed in this article are solely those of the authors and do not necessarily represent those of their affiliated organizations, or those of the publisher, the editors and the reviewers. Any product that may be evaluated in this article, or claim that may be made by its manufacturer, is not guaranteed or endorsed by the publisher.

## References

[1] Feigin V Roth G Naghavi M Parmar P Krishnamurthi R Chugh S. Global burden of diseases, injuries and risk factors study 2013 and stroke experts writing group. global burden of stroke and risk factors in 188 countries, during 1990-2013: a systematic analysis for the global burden of disease study 2013. Lancet Neurol. (2016) 15:913–24. 10.1016/S1474-4422(16)30073-4 27291521

[2] Eyding J Bartig D Weber R Katsanos AH Weimar C Hacke W. Inpatient TIA and stroke care in adult patients in Germany-retrospective analysis of nationwide administrative data sets of 2011 to 2017. Neurol Res Pract. (2019) 1:1–8. 10.1186/s42466-019-0044-y 33324904 PMC7650112

[3] Icks A Claessen H Kvitkina T Narres M Weingärtner M Schwab S. Incidence and relative risk of stroke in the diabetic and the non-diabetic population between 1998 and 2014: a community-based stroke register. PLoS ONE. (2017) 12:e0188306. 10.1371/journal.pone.0188306 29145522 PMC5690660

[4] Rücker V Wiedmann S O'Flaherty M Busch MA Heuschmann PU. Decline in regional trends in mortality of stroke subtypes in Germany from 1998 to 2015. Stroke. (2018) 49:2577–83. 10.1161/STROKEAHA.118.023193 30355214

[5] Lawrence ES Coshall C Dundas R Stewart J Rudd AG Howard R. Estimates of the prevalence of acute stroke impairments and disability in a multiethnic population. Stroke. (2001) 32:1279–84. 10.1161/01.STR.32.6.1279 11387487

[6] Coupar F Pollock A Rowe P Weir C Langhorne P. Predictors of upper limb recovery after stroke: a systematic review and meta-analysis. Clin Rehabil. (2012) 26:291–313. 10.1177/0269215511420305 22023891

[7] Kwakkel G Kollen BJ van der Grond J Prevo AJ. Probability of regaining dexterity in the flaccid upper limb: impact of severity of paresis and time since onset in acute stroke. Stroke. (2003) 34:2181–6. 10.1161/01.STR.0000087172.16305.CD 12907818

[8] Heller A Wade DT Wood VA Sunderland A Hewer RL Ward E. Arm function after stroke: measurement and recovery over the first three months. J Neurol Neurosurg Psychiatry. (1987) 50:714–9. 10.1136/jnnp.50.6.714 3612152 PMC1032076

[9] Wade D Langton-Hewer R Wood VA Skilbeck C Ismail H. The hemiplegic arm after stroke: measurement and recovery. J Neurol Neurosurg Psychiatry. (1983) 46:521–4. 10.1136/jnnp.46.6.521 6875585 PMC1027442

[10] Veerbeek JM Kwakkel G van Wegen EE Ket JC Heymans MW. Early prediction of outcome of activities of daily living after stroke: a systematic review. Stroke. (2011) 42:1482–8. 10.1161/STROKEAHA.110.604090 21474812

[11] Etoom M Hawamdeh M Hawamdeh Z Alwardat M Giordani L Bacciu S. Constraint-induced movement therapy as a rehabilitation intervention for upper extremity in stroke patients: systematic review and meta-analysis. Int J Rehabil Res. (2016) 39:197–210. 10.1097/MRR.0000000000000169 27123790

[12] Langhorne P Coupar F Pollock A. Motor recovery after stroke: a systematic review. Lancet Neurol. (2009) 8:741–54. 10.1016/S1474-4422(09)70150-4 19608100

[13] Veerbeek JM van Wegen E van Peppen R van der Wees PJ Hendriks E Rietberg M. What is the evidence for physical therapy poststroke? a systematic review and meta-analysis. PLoS ONE. (2014) 9:e87987. 10.1371/journal.pone.0087987 24505342 PMC3913786

[14] Wolf SL Blanton S Baer H Breshears J Butler AJ. Repetitive task practice: a critical review of constraint-induced movement therapy in stroke. Neurologist. (2002) 8:325. 10.1097/00127893-200211000-00001 12801434 PMC3572508

[15] Pollock A Farmer SE Brady MC Langhorne P Mead GE Mehrholz J. Interventions for improving upper limb function after stroke. Cochrane Database Syst Rev. (2014) 2014:CD010820. 10.1002/14651858.CD010820.pub2 25387001 PMC6469541

[16] Thieme H Mehrholz J Pohl M Behrens J Dohle C. Mirror therapy for improving motor function after stroke. Stroke. (2013) 44:e1–2. 10.1161/STROKEAHA.112.673087 23390640

[17] Platz T Fheodoroff K Mehrholz J. S3 guideline Rehabilitation therapy for Arm Paresis After Stroke of the DGNR Long Version. (2020). Available online at: https://www.awmf.org/uploads/tx_szleitlinien/080-001l_S3_Rehabilitative_Therapie_bei_Armparese_nach_Schlaganfall_2020-07.pdf

[18] Platz T Eickhof C Van Kaick S Engel U Pinkowski C Kalok S. Impairment-oriented training or bobath therapy for severe arm paresis after stroke: a single-blind, multicentre randomized controlled trial. Clin Rehabil. (2005) 19:714–24. 10.1191/0269215505cr904oa 16250190

[19] Urton ML Kohia M Davis J Neill MR. Systematic literature review of treatment interventions for upper extremity hemiparesis following stroke. Occup Ther Int. (2007) 14:11–27. 10.1002/oti.220 17623376

[20] Mehrholz J Pohl M Platz T Kugler J Elsner B. Electromechanical and robot-assisted arm training for improving activities of daily living, arm function, and arm muscle strength after stroke. Cochrane Database Syst Rev. (2018) 9:CD006876. 10.1002/14651858.CD006876.pub5 30175845 PMC6513114

[21] Zhang C Li-Tsang CW Au RK. Robotic approaches for the rehabilitation of upper limb recovery after stroke: a systematic review and meta-analysis. Int J Rehabil Res. (2017) 40:19–28. 10.1097/MRR.0000000000000204 27926617

[22] Kollen BJ Lennon S Lyons B Wheatley-Smith L Scheper M Buurke JH. The effectiveness of the bobath concept in stroke rehabilitation: what is the evidence? Stroke. (2009) 40:e89–97. 10.1161/STROKEAHA.108.533828 19182079

[23] Winter J Hunter S Sim J Crome P. Hands-on therapy interventions for upper limb motor dysfunction following stroke. Cochrane Database Syst Rev. (2011) 2011:CD006609. 10.1002/14651858.CD006609.pub2 21678359 PMC6464865

[24] Hesse S Mehrholz J Werner C. Robot-assisted upper and lower limb rehabilitation after stroke: walking and arm/hand function. Dtsch Ärztebl Int. (2008) 105:330. 10.3238/arztebl.2008.0330 19629252 PMC2707632

[25] Kwakkel G Kollen BJ Krebs HI. Effects of robot-assisted therapy on upper limb recovery after stroke: a systematic review. Neurorehabil Neural Repair. (2008) 22:111–21. 10.1177/1545968307305457 17876068 PMC2730506

[26] Wu C-Y Huang P-C Chen Y-T Lin K-C Yang H-W. Effects of mirror therapy on motor and sensory recovery in chronic stroke: a randomized controlled trial. Arch Phys Med Rehabil. (2013) 94:1023–30. 10.1016/j.apmr.2013.02.007 23419791

[27] Birbaumer N Cohen LG. Brain-computer interfaces: communication and restoration of movement in paralysis. J Physiol. (2007) 579:621–36. 10.1113/jphysiol.2006.125633 17234696 PMC2151357

[28] Van Dokkum L Ward T Laffont I. Brain computer interfaces for neurorehabilitation–its current status as a rehabilitation strategy post-stroke. Ann Phys Rehabil Med. (2015) 58:3–8. 10.1016/j.rehab.2014.09.016 25614021

[29] Soekadar SR Witkowski M Mellinger J Ramos A Birbaumer N Cohen LG. ERD-based online brain-machine interfaces (BMI) in the context of neurorehabilitation: optimizing BMI learning and performance. IEEE Trans Neural Syst Rehabil Eng. (2011) 19:542–9. 10.1109/TNSRE.2011.2166809 21984519 PMC4884650

[30] Buch E Weber C Cohen LG Braun C Dimyan MA Ard T. Think to move: a neuromagnetic brain-computer interface (BCI) system for chronic stroke. Stroke. (2008) 39:910–7. 10.1161/STROKEAHA.107.505313 18258825 PMC5494966

[31] Soekadar SR Birbaumer N Slutzky MW Cohen LG. Brain-machine interfaces in neurorehabilitation of stroke. Neurobiol Dis. (2015) 83:172–9. 10.1016/j.nbd.2014.11.025 25489973

[32] Mrachacz-Kersting N Jiang N Stevenson AJT Niazi IK Kostic V Pavlovic A. Efficient neuroplasticity induction in chronic stroke patients by an associative brain-computer interface. J Neurophysiol. (2016) 115:1410–21. 10.1152/jn.00918.2015 26719088 PMC4808132

[33] Ushiba J Soekadar SR. Brain-machine interfaces for rehabilitation of poststroke hemiplegia. Prog Brain Res. (2016) 228:163–83. 10.1016/bs.pbr.2016.04.020 27590969

[34] Laffont I Bakhti K Coroian F Van Dokkum L Mottet D Schweighofer N. Innovative technologies applied to sensorimotor rehabilitation after stroke. Ann Phys Rehabil Med. (2014) 57:543–51. 10.1016/j.rehab.2014.08.007 25261273

[35] Pichiorri F Morone G Petti M Toppi J Pisotta I Molinari M. Brain-computer interface boosts motor imagery practice during stroke recovery. Ann Neurol. (2015) 77:851–65. 10.1002/ana.24390 25712802

[36] Ang KK Chua KSG Phua KS Wang C Chin ZY Kuah CWK. A randomized controlled trial of EEG-based motor imagery brain-computer interface robotic rehabilitation for stroke. Clin EEG Neurosci. (2015) 46:310–20. 10.1177/1550059414522229 24756025

[37] Biasiucci A Leeb R Iturrate I Perdikis S Al-Khodairy A Corbet T. Brain-actuated functional electrical stimulation elicits lasting arm motor recovery after stroke. Nat Commun. (2018) 9:1–13. 10.1038/s41467-018-04673-z 29925890 PMC6010454

[38] Broetz D Braun C Weber C Soekadar SR Caria A Birbaumer N. Combination of brain-computer interface training and goal-directed physical therapy in chronic stroke: a case report. Neurorehabil Neural Repair. (2010) 24:674–9. 10.1177/1545968310368683 20519741

[39] Ramos-Murguialday A Broetz D Rea M Laer L Yilmaz O Brasil FL. Brain-machine interface in chronic stroke rehabilitation: a controlled study. Ann Neurol. (2013) 74:100–8. 10.1002/ana.23879 23494615 PMC3700597

[40] Coscia M Wessel MJ Chaudary U Millán JdR Micera S Micera S. Neurotechnology-aided interventions for upper limb motor rehabilitation in severe chronic stroke. Brain. (2019) 142:2182–97. 10.1093/brain/awz181 31257411 PMC6658861

[41] Hömberg V. Neurorehabilitation approaches to facilitate motor recovery. Handb Clin Neurol. (2013) 110:161–73. 10.1016/B978-0-444-52901-5.00014-9 23312639

[42] Knecht S Hesse S Oster P. Rehabilitation after stroke. Dtsch Ärztebl Int. (2011) 108:600. 10.3238/arztebl.2011.0600 21966318 PMC3183303

[43] Bertram M Brandt T. Early neurological-neurosurgical rehabilitation. current state. Nervenarzt. (2007) 78:1160–74. 10.1007/s00115-007-2269-1 17457558

[44] Mihara M Hattori N Hatakenaka M Yagura H Kawano T Hino T. Near-infrared spectroscopy-mediated neurofeedback enhances efficacy of motor imagery-based training in poststroke victims: a pilot study. Stroke. (2013) 44:1091–8. 10.1161/STROKEAHA.111.674507 23404723

[45] Ono T Shindo K Kawashima K Ota N Ito M Ota T. Brain-computer interface with somatosensory feedback improves functional recovery from severe hemiplegia due to chronic stroke. Front Neuroeng. (2014) 7:19. 10.3389/fneng.2014.00019 25071543 PMC4083225

[46] Ono T Kimura A Ushiba J. Daily training with realistic visual feedback improves reproducibility of event-related desynchronisation following hand motor imagery. Clin Neurophysiol. (2013) 124:1779–86. 10.1016/j.clinph.2013.03.006 23643578

[47] Frolov AA Húsek Da Biryukova EV Bobrov PD Mokienko OA. Principles of motor recovery in post-stroke patients using hand exoskeleton controlled by the brain-computer interface based on motor imagery. Neural Netw World. (2017) 27:107. 10.14311/NNW.2017.27.006

[48] Nishimoto A Kawakami M Fujiwara T Hiramoto M Honaga K Abe K. Feasibility of task-specific brain-machine interface training for upper-extremity paralysis in patients with chronic hemiparetic stroke. J Rehabil Med. (2018) 50:52–8. 10.2340/16501977-2275 28949370

[49] Gaete JM Bogousslavsky J. Post-stroke depression. Expert Rev Neurother. (2008) 8:75–92. 10.1586/14737175.8.1.75 18088202

[50] Bundy DT Souders L Baranyai K Leonard L Schalk G Coker R. Contralesional brain-computer interface control of a powered exoskeleton for motor recovery in chronic stroke survivors. Stroke. (2017) 48:1908–15. 10.1161/STROKEAHA.116.016304 28550098 PMC5482564

[51] Soekadar SR Nann M Crea S Trigili E Gómez C Opisso E. Restoration of finger and arm movements using hybrid brain/neural assistive technology in everyday life environments. In: Christoph Guger NM-K, Allison BZ, Editors. Brain-Computer Interface Research, A State-of-the-Art Summary. Basel: Springer International Publishing (2019). p. 53–61. 10.1007/978-3-030-05668-1_5

[52] Serruya MD. Bottlenecks to clinical translation of direct brain-computer interfaces. Front Syst Neurosci. (2014) 8:226. 10.3389/fnsys.2014.00226 25520632 PMC4251316

[53] Witkowski M Cortese M Cempini M Mellinger J Vitiello N Soekadar SR. Enhancing brain-machine interface (BMI) control of a hand exoskeleton using electrooculography (EOG). J Neuroeng Rehabil. (2014) 11:165. 10.1186/1743-0003-11-165 25510922 PMC4274709

[54] Colamarino E de Seta V Masciullo M Cincotti F Mattia D Pichiorri F. Corticomuscular and intermuscular coupling in simple hand movements to enable a hybrid brain–computer interface. Int J Neural Syst. (2021) 31:2150052. 10.1142/S0129065721500520 34590990

[55] Cavallo A Roth V Haslacher D Nann M Soekadar SR. Minimizing bio-signal recording sites for noninvasive hybrid brain/neural control. IEEE Syst J. (2020) 15:1540–6. 10.1109/JSYST.2020.302175127295638

[56] Kam JW Griffin S Shen A Patel S Hinrichs H Heinze H-J. Systematic comparison between a wireless EEG system with dry electrodes and a wired EEG system with wet electrodes. Neuroimage. (2019) 184:119–29. 10.1016/j.neuroimage.2018.09.012 30218769 PMC6568010

[57] Blankertz B Sannelli C Halder S Hammer EM Kübler A Müller K-R. Neurophysiological predictor of SMR-based BCI performance. Neuroimage. (2010) 51:1303–9. 10.1016/j.neuroimage.2010.03.022 20303409

[58] Vidaurre C Sannelli C Müller K-R Blankertz B. Co-adaptive calibration to improve BCI efficiency. J Neural Eng. (2011) 8:025009. 10.1088/1741-2560/8/2/025009 21436515

[59] Thompson MC. Critiquing the concept of BCI illiteracy. Sci Eng Ethics. (2019) 25:1217–33. 10.1007/s11948-018-0061-1 30117107

[60] Lotte F Larrue F Mühl C. Flaws in current human training protocols for spontaneous brain-computer interfaces: lessons learned from instructional design. Front Hum Neurosci. (2013) 7:568. 10.3389/fnhum.2013.00568 24062669 PMC3775130

[61] Prasad G Herman P Coyle D McDonough S Crosbie J. Applying a brain-computer interface to support motor imagery practice in people with stroke for upper limb recovery: a feasibility study. J Neuroeng Rehabil. (2010) 7:60. 10.1186/1743-0003-7-60 21156054 PMC3017056

[62] Hammer EM Halder S Blankertz B Sannelli C Dickhaus T Kleih S. Psychological predictors of SMR-BCI performance. Biol psychol. (2012) 89:80–6. 10.1016/j.biopsycho.2011.09.006 21964375

[63] Myrden A Chau T. Effects of user mental state on EEG-BCI performance. Front Hum Neurosci. (2015) 9:308. 10.3389/fnhum.2015.00308 26082705 PMC4451337

[64] Nann M Haslacher D Colucci A Eskofier B von Tscharner V Soekadar SR. Heart rate variability predicts decline in sensorimotor rhythm control. J Neural Eng. (2021) 18:0460b5. 10.1088/1741-2552/ac1177 34229308

[65] Fels M Bauer R Gharabaghi A. Predicting workload profiles of brain–robot interface and electromygraphic neurofeedback with cortical resting-state networks: personal trait or task-specific challenge? J Neural Eng. (2015) 12:046029. 10.1088/1741-2560/12/4/046029 26170164

[66] Lorenz R Pascual J Blankertz B Vidaurre C. Towards a holistic assessment of the user experience with hybrid BCIs. J Neural Eng. (2014) 11:035007. 10.1088/1741-2560/11/3/035007 24835132

[67] Guggisberg AG Koch PJ Hummel FC Buetefisch CM. Brain networks and their relevance for stroke rehabilitation. Clin Neurophysiol. (2019) 130:1098–124. 10.1016/j.clinph.2019.04.004 31082786 PMC6603430

[68] Ruddy K Balsters J Mantini D Liu Q Kassraian-Fard P Enz N. Neural activity related to volitional regulation of cortical excitability. Elife. (2018) 7:e40843. 10.7554/eLife.40843.022 30489255 PMC6294548

[69] Raffin E Hummel FC. restoring motor functions after stroke: multiple approaches and opportunities. Neuroscientist. (2017) 24:400–16. 10.1177/1073858417737486 29283026

[70] Van Meer MP Van Der Marel K Wang K Otte WM El Bouazati S Roeling TA. Recovery of sensorimotor function after experimental stroke correlates with restoration of resting-state interhemispheric functional connectivity. J Neurosci. (2010) 30:3964–72. 10.1523/JNEUROSCI.5709-09.2010 20237267 PMC6632290

[71] Westlake KP Hinkley LB Bucci M Guggisberg AG Findlay AM Henry RG. Resting state alpha-band functional connectivity and recovery after stroke. Exp Neurol. (2012) 237:160–9. 10.1016/j.expneurol.2012.06.020 22750324 PMC3646713

[72] Sannelli C Vidaurre C Muller KR Blankertz B. A large scale screening study with a SMR-based BCI: categorization of BCI users and differences in their SMR activity. PLoS ONE. (2019) 14:e0207351. 10.1371/journal.pone.0207351 30682025 PMC6347432

